# Rethinking broad consent in the age of genomic AI: the Hungarian case

**DOI:** 10.3389/fmed.2026.1936581

**Published:** 2026-09-30

**Authors:** Mónika Nogel

**Affiliations:** Department of Criminology and Forensic Sciences, Deák Ferenc Faculty of Law and Political Sciences, Széchenyi István University, Győr, Hungary

**Keywords:** artificial intelligence, biobanks, broad consent, precision medicine, research ethics

## Introduction

Broad consent enables participants to authorize the use of their biosamples and health data across multiple future medical studies whose precise aims cannot all be specified at enrollment. It supports precision medicine by making large, diverse, and longitudinal datasets available for research on disease risk, diagnosis, prevention, and treatment response. In this article, AI-assisted genomic analysis refers to the use of computational models, including machine-learning systems, to identify, classify, prioritize, or reinterpret genomic variants and associated health information in research and translational settings. Stored genomic data can therefore be reanalyzed repeatedly as models, reference datasets, variant classifications, and scientific knowledge evolve.

Here, an inference is an interpretive output derived from genomic or linked health data, such as a variant classification, disease-risk estimate, or treatment-response prediction. Such an output may acquire clinical relevance long after the original sample was collected. Hungary's 2025 reform brings this temporal problem into focus. It permits broad consent for current and future medical research of “*national strategic significance,”* yet provides no mechanism within the research framework for returning individual findings or re-identifying coded material for the participant's benefit. The framework gives research institutions substantial future flexibility and fixes the participant's informational position at the time of consent. This article describes the distance between the material initially contributed and the later interpretive outputs as the sample–data–inference gap.

The analysis first sets out the current Hungarian framework and identifies where its categorical non-return rule falls short. It then distinguishes binding European Union law from non-binding European guidance and national comparators, explains why AI-assisted genomic reanalysis intensifies the problem, and proposes a model of bounded and reciprocal broad consent. The model combines defined consent boundaries, ongoing transparency, review triggered by material changes, and a limited pathway for independently validated, clinically actionable findings.

## The Hungarian framework for broad consent

Act XXII of 2025 introduced the new framework through amendments to the Hungarian Health Act (1). Broad consent now covers biological samples and associated data used in authorized and subsequently authorized medical research of “*national strategic significance”.* The statutory category includes research aimed at improving population health, supporting early detection, examining major causes of mortality, and increasing treatment effectiveness. Consent must be voluntary and may be withdrawn. Samples and data are stored in coded form, and each later project remains subject to authorization and ethical review (2). The treatment of individual findings creates the central difficulty.

Participants must be told before consenting that they will receive no information about research results. The implementing decree also excludes re-identification of coded samples or data for the benefit of the participant or a close relative (3).

## Limits of the categorical non-return rule

The treatment of individual findings creates the central difficulty. Read together, these provisions leave no express route within the research framework for communicating a finding that later becomes serious, independently confirmed, and clinically actionable. Confidentiality, the separation of research from clinical care, and the risks of communicating uncertain findings provide legitimate reasons for caution. A complete closure of the feedback pathway requires a separate justification.

Empirical research also cautions against treating a signed form as acceptance of every condition within the consent package. In a large German study, a better understanding of broad consent was associated with both a greater willingness to participate and a stronger opposition to a strict non-reporting policy. Despite disagreeing with the withholding of potentially relevant findings, many patients nevertheless supported the research (4). A systematic review from the USA similarly found that support for broad consent was often higher when it was the only available option, and fell when participants were offered more granular alternatives or when commercial access was contemplated (5). An initial signature may validly record a choice not to receive future information if that choice and its reasonably foreseeable implications are adequately explained. It cannot, however, demonstrate acceptance of informational consequences that were unknown or not reasonably foreseeable at the time of consent.

## European legal and governance context

The relevant European instruments differ in legal force. The General Data Protection Regulation (GDPR) is binding in its entirety and directly applicable in Hungary. The Charter of Fundamental Rights of the European Union binds Hungary when it is implementing Union law. The Oviedo Convention is binding on Hungary as a Contracting Party. By contrast, the version of European Data Protection Board (EDPB) Guidelines 1/2026 adopted for public consultation provides non-binding interpretive guidance, while Recommendation CM/Rec(2016)6 provides non-binding policy guidance. The European Health Data Space Regulation (EHDS) is likewise a binding EU regulation, although Chapter IV on the secondary use of electronic health data will apply from 26 March 2029.

GDPR Recital 33 acknowledges that the purposes of scientific research cannot always be fully identified at the time of data collection (6). This flexibility concerns purposes that are genuinely not yet known; it should not be used to leave known or reasonably foreseeable purposes unspecified. The current version of EDPB Guidelines 1/2026, adopted for public consultation, treats this flexibility as limited. The research domain must remain clearly defined, transparency must be maintained, and the right to withdraw must remain effective (7). The EDPB further distinguishes consent to participate in research, arising from ethical or sector-specific requirements, from consent used as a legal basis under the GDPR. The former concerns whether the person agrees to take part in the research. Separately, the controller must identify a lawful basis for processing under Article 6 and, because genomic and health data are special-category data, an applicable condition under Article 9. These may coincide where GDPR consent is relied upon, but they are not automatically the same.

Article 15 of the GDPR addresses access to personal data that the controller has already processed. It does not impose a general obligation to establish clinical services, validate all research outputs, or convert algorithmic scores into clinical diagnoses. An inference that is retained may still constitute personal data if it remains associated with an identifiable participant. Therefore, its data protection status and its suitability for clinical reporting require distinct analyses. Accordingly, GDPR compliance does not itself create a clinical return-of-findings service; the concern addressed here is the absence of a separate mediated pathway under Hungarian research law.

European bioethics instruments introduce an autonomy-based dimension. Article 10 of the Oviedo Convention safeguards individuals’ interests in accessing health-related information and in choosing to remain uninformed (8). Recommendation CM/Rec(2016)6 calls for transparent governance of biological materials used in research and an appropriate feedback policy (9). Together, these instruments reinforce participant autonomy when research findings attain clinical relevance.

The EHDS establishes a future institutional model rather than a presently operational remedy. Under its secondary-use framework, health data users may not re-identify individuals. Where a health data user reports a significant finding, the health data access body will inform the health data holder, which will inform the individual or the treating professional in accordance with national law. Individuals retain the right to request not to be informed. This mediated approach maintains a clear distinction between research and clinical care, while allowing a limited pathway for findings of substantial health relevance (10).

Article 52 of the Charter supplies the proportionality framework when the Hungarian scheme operates within the scope of EU law (11). The restrictions serve legitimate purposes by reducing the risk of re-identification, limiting the communication of uncertain results, and maintaining the distinction between research and clinical care.

While these measures are suitable for these objectives, establishing their necessity is more challenging. The relevant comparator is Hungary's complete closure of the feedback pathway, not the EHDS mechanism itself. Compared with complete closure, a mediated route preserves the participant's option to receive or refuse a qualifying finding without allowing researchers direct access to identity. Independent clinical review, confirmatory testing, a high threshold for seriousness and actionability, and documented participant preferences would determine which findings enter that route. These safeguards supplement, rather than duplicate, the EHDS model. The evaluation should therefore distinguish between the broad-consent framework and the complete closure of the feedback pathway. Broad consent may remain justified if appropriate safeguards are implemented. However, the categorical exclusion of any return mechanism demands a more robust justification.

The German Medical Informatics Initiative, although a national standard rather than legislation, already combines project-level review, public transparency, separate recontact options, and exceptional feedback without giving researchers direct access to identities (12). Norwegian guidance likewise confines broad consent to defined research fields and requires continuing information, with renewed consent only when later use falls outside the original scope (13). These comparators are not exact analogs of the Hungarian legislation, but they weaken the claim that categorical non-return is the least restrictive means available.

## Why AI-assisted genomic reanalysis matters for broad consent

AI-assisted genomic analysis intensifies the sample–data–inference gap because the same stored sequences may be reanalyzed as reference databases expand, variant classifications evolve, and models are retrained or recalibrated. Automated systems can generate candidate findings at scale long after initial data collection. Consequently, governance frameworks must address the management of samples and records, and the inferences derived from stored data.

AI-generated outputs require careful interpretation before they can inform clinical care. Their reliability depends on the model version, the training and reference datasets, the decision threshold, and the validation populations. Expert assessment, independent confirmation, and evidence of clinical validity and actionability remain essential (14). An exploratory inference may later acquire sufficient evidential support for clinical use. The regulatory framework should allow a fresh assessment when that threshold is reached. Broad consent to research use should not automatically be treated as advance authorization for every later clinical evaluation or communication of an AI-derived finding.

Documentation of any candidate finding should include the model version, validation population, and decision threshold. The EHDS explicitly encompasses inferred, derived, and automatically generated health data, and permits secondary use for training, testing, and evaluating algorithms and AI systems. Transitioning from research use to clinical decision support should prompt a new ethical and regulatory review. The same requirement applies when the intended purpose of a model undergoes a significant change. Reviews should also assess performance across relevant populations, as under-representation in genomic databases and training datasets may reduce accuracy and perpetuate existing inequalities (15). These developments do not automatically require project-specific reconsent, but they should trigger a review of whether the use remains within the original consent boundaries and whether updated information or a new participant choice is required.

## Proposed reform: bounded and reciprocal broad consent

Hungary does not require project-specific reconsent for every new analysis. Requiring this would create a heavy burden, leave out participants who cannot be reached, and could lead to selection bias. A better approach is to use, “*bounded and reciprocal broad consent”*.

‘*Bounded*’ means that future research stays within clear limits. Consent materials should describe, at an appropriate level of specificity, the relevant research fields, data types, linkage practices, commercial involvement, cross-border transfers, and reasonably foreseeable AI uses, such as model training and automated reanalysis. These categories define the scope of authorization and the information owed to participants. Future uses cannot all be predicted at enrollment; major changes, such as moving into a new research area, using a new data source, changing the main purpose of a model, or starting clinical use, should trigger renewed review and, where appropriate, updated information or a new participant choice.

‘*Reciprocal*’ means that while research can be flexible, there are ongoing responsibilities. Approved projects should be listed in a public register before using any data, with clear information about their goals, data types, partners, funding, and AI functions. Participants should have the option to review and update their preferences, including whether they wish to be contacted about important findings. Recording this preference is one condition of a return pathway; a finding must also satisfy the relevant clinical and governance criteria and recontact must occur through an authorized institutional route. Recent studies show that transparency, control, timing, and how accurately consent is recorded are just as important as the wording of the consent form (16, 17).

Within this proposed model, the safeguards identified above would operate through a limited return pathway. Researchers should neither re-identify participants directly nor communicate raw model outputs. An independent multidisciplinary team should determine whether an already identified finding is serious, has been independently confirmed, is clinically valid and actionable, and accords with the participant's recorded preference. Recontact should occur through a trusted intermediary or the participant's doctor, with genetic counseling where appropriate. The responsibility is limited to the assessment and careful handling of qualifying findings that emerge during the research and does not extend to continuous searching for every possible result.

## Discussion

Hungary's reform addresses the need for stable and efficient access to biological samples and health data for long-term public-interest research. The broad-consent framework is designed to facilitate this objective. Under the current rules, the participant's informational position is fixed before the future significance of genomic data can be fully understood.

AI-assisted genomic reanalysis challenges the sustainability of this fixed informational arrangement. As analytical models, reference datasets, and scientific knowledge advance, stored data may yield new clinically relevant inferences. Hungarian law should maintain a presumption against returning unvalidated research results and establish a distinct, mediated process for communicating findings that satisfy predefined criteria for seriousness, independent confirmation, clinical validity, and actionability. A broader initial authorization should correspond to heightened ongoing obligations regarding transparency, oversight, participant autonomy, and reciprocity.
